# Treatment for First Cytomegalovirus Infection Post–Hematopoietic Cell Transplant in the AURORA Trial: A Multicenter, Double-Blind, Randomized, Phase 3 Trial Comparing Maribavir With Valganciclovir

**DOI:** 10.1093/cid/ciad709

**Published:** 2023-11-30

**Authors:** Genovefa A Papanicolaou, Robin K Avery, Catherine Cordonnier, Rafael F Duarte, Shariq Haider, Johan Maertens, Karl S Peggs, Carlos Solano, Jo-Anne H Young, Martha Fournier, Rose Ann Murray, Jingyang Wu, Drew J Winston, AURORA Trial Investigators, Deepak Singhal, Joe Sasadeusz, Johan Maertans, Aspasia Georgala, Dominik Selleslag, Anke Verlinden, Tessa Kerre, Ann De Becker, Shariq Haider, Alissa Wright, Depei Wu, Radovan Vrhovac, Catherine Cordonnier, Ana Berceanu, Sylvie Francois, David Michonneau, Anne Huynh, Wolfgang Bethge, Martin Kaufmann, Matthias Stelljes, Georg-Nikolaus Franke, Timo Schmitt, Lutz Müller, Manfred Ahlgrimm, Judith Niederland, Panagiotis Tsirigotis, Ron Ram, Noga Shemtov, Tsila Rosenvald-Zuckerman, Ilaria Cutini, Alessandro Busca, Francesco Onida, Cristina Tecchio, Peter Browett, Young Rok Do, Sung Hyun Kim, Aloysius Ho, Liang Piu Koh, Maria Lourdes Vazquez Lopez, Javier Lopez Jimenez, Christelle Ferra Coll, Rafael De la Camara, Carlos Solano, Alberto Mussetti, Juan Carlos Vallejo Llamas, Pere Barba Suñol, Manuel Jurado Chacón, Rafael F Duarte, María Aranzazu Bermúdez Rodríguez, Nicolas Mueller, Hakan Ozdogu, Gunhan Gurman, Adrian Bloor, Bhuvan Kishore, Kari S Peggs, Dragana Milojkovic, Kim Orchard, Arpad Gabor Toth, Mickey Koh, Robin K Avery, Jennifer Pisano, George Alangaden, Drew J Winston, Genovefa Papanicolau, Benjamin Gewurz, Francisco M Marty, Jo-Anne H Young, Patrick Hagen, Ran Reshef, Sameem Abedin, Paul Shaughnessy, Laura Gibson, Joan Tsiporah Shore, Carlos R Bachier, Jean Yared, Maricar Malinis

**Affiliations:** Memorial Sloan Kettering Cancer Center, New York, New York, USA; Johns Hopkins University, Baltimore, Maryland, USA; Henri Mondor Hôpital, Assistance Publique-Hopitaux de Paris, and Université Paris-Est-Créteil, Créteil, France; Hospital Universitario Puerta de Hierro Majadahonda, Madrid, Spain; Hamilton Health Sciences Corporation, Ontario, Canada; University Hospitals Leuven, KU Leuven, Leuven, Belgium; University College London Hospitals NHS Foundation Trust, London, United Kingdom; Hospital Clínico Universitario, University of Valencia, Valencia, Spain; University of Minnesota, Minneapolis, Minnesota, USA; Takeda Development Center Americas, Inc, Lexington, Massachusetts, USA; Takeda Development Center Americas, Inc, Lexington, Massachusetts, USA; Takeda Development Center Americas, Inc, Lexington, Massachusetts, USA; Los Angeles Medical Center, University of California, Los Angeles, California, USA

**Keywords:** cytomegalovirus, hematopoietic cell transplant, maribavir, valganciclovir

## Abstract

**Background:**

Neutropenia may limit the use of valganciclovir treatment for cytomegalovirus (CMV) infection following hematopoietic cell transplant (HCT). A phase 2 study indicated efficacy of maribavir with fewer treatment-limiting toxicities than valganciclovir.

**Methods:**

In this multicenter, double-blind, phase 3 study, patients with first asymptomatic CMV infection post-HCT were stratified and randomized 1:1 to maribavir 400 mg twice daily or valganciclovir (dose-adjusted for renal clearance) for 8 weeks with 12 weeks of follow-up. The primary endpoint was confirmed CMV viremia clearance at week 8 (primary hypothesis of noninferiority margin of 7.0%). The key secondary endpoint was a composite of the primary endpoint with no findings of CMV tissue-invasive disease at week 8 through week 16. Treatment-emergent adverse events (TEAEs) were assessed.

**Results:**

Among patients treated (273 maribavir; 274 valganciclovir), the primary endpoint of noninferiority of maribavir was not met (maribavir, 69.6%; valganciclovir, 77.4%; adjusted difference: −7.7%; 95% confidence interval [CI]: −14.98, −.36; lower limit of 95% CI of treatment difference exceeded −7.0%). At week 16, 52.7% and 48.5% of patients treated (maribavir and valganciclovir, respectively) maintained CMV viremia clearance without tissue-invasive disease (adjusted difference: 4.4%; 95% CI: −3.91, 12.76). With maribavir (vs valganciclovir), fewer patients experienced neutropenia (16.1% and 52.9%) or discontinued due to TEAEs (27.8% and 41.2%). Discontinuations were mostly due to neutropenia (maribavir, 4.0%; valganciclovir, 17.5%).

**Conclusions:**

Although noninferiority of maribavir to valganciclovir for the primary endpoint was not achieved based on the prespecified noninferiority margin, maribavir demonstrated comparable CMV viremia clearance during post-treatment follow-up, with fewer discontinuations due to neutropenia.

**Clinical Trials Registration.** NCT02927067 [AURORA].

## Teaser

Noninferiority of maribavir to valganciclovir was not met for the primary endpoint of CMV viremia clearance at study week 8. However, maribavir had comparable post-treatment CMV viremia clearance to valganciclovir, and was associated with a lower incidence of treatment-limiting neutropenia.

## Graphical Abstract

**Graphical Abstract:**
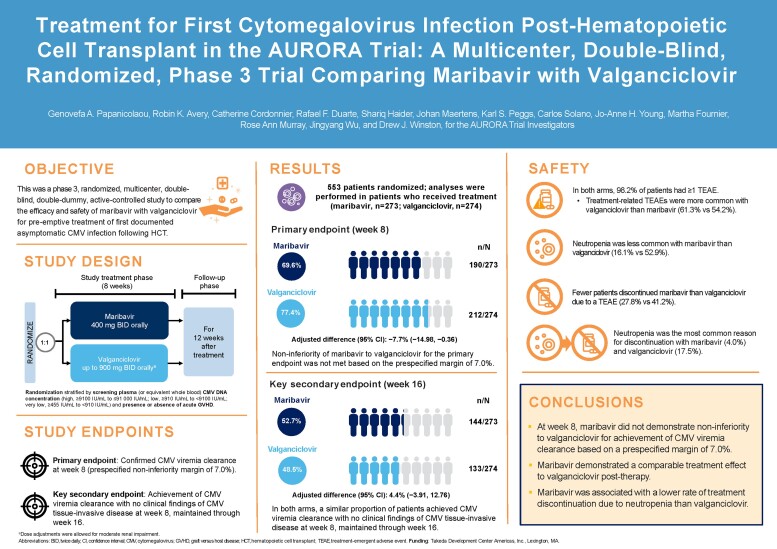

Cytomegalovirus (CMV) is a common cause of morbidity for hematopoietic cell transplant (HCT) recipients [1–4]. Pre-emptive treatment for CMV following HCT is generally effective in preventing CMV tissue-invasive disease (ie, end-organ disease) but is associated with dose-limiting toxicities (eg, myelotoxicity for valganciclovir/ganciclovir and renal toxicity for foscarnet and cidofovir [5–9]), often requiring dose adjustment, interruption, or cycling of therapies, thereby impacting CMV recurrence and patient outcomes [10, 11]. Additionally, management of treatment-related toxicities with growth-factor support, hydration, intensive electrolyte monitoring, and replacement poses a substantial burden on both the healthcare system and patients [3, 10]. Therefore, there is a need for new agents with fewer safety concerns for CMV management in HCT recipients.

Maribavir, a benzimidazole riboside, is an orally bioavailable antiviral [12] with multimodal anti-CMV activity through the inhibition of CMV DNA replication, encapsidation, and nuclear egress of viral capsids via inhibition of CMV-specific UL97 protein kinase [13–15]. Maribavir demonstrated in vitro activity against CMV, including strains resistant to ganciclovir, foscarnet, or cidofovir [16]. In the SOLSTICE trial (NCT02931539) for refractory CMV infections, maribavir 400 mg twice daily (BID) demonstrated superiority to investigator-assigned anti-CMV treatment for CMV viremia clearance at week 8 and maintained CMV viremia clearance and CMV infection symptom control through week 16 [6]. Maribavir has been approved for the treatment of post-transplant CMV infection or disease refractory (with or without resistance, with or without intolerance) to treatment with ganciclovir, valganciclovir, foscarnet, or cidofovir in various countries [6]. A phase 2 study indicated efficacy of maribavir in CMV viremia clearance compared with valganciclovir for pre-emptive treatment of first CMV infection following HCT and solid-organ transplantation [17]. In both trials, maribavir was associated with a lower incidence of neutropenia than valganciclovir/ganciclovir [6, 17].

The phase 3 AURORA trial (NCT02927067) was designed to compare the efficacy and safety of maribavir with valganciclovir for pre-emptive treatment of the first asymptomatic CMV infection following HCT, with a hypothesis of noninferiority of maribavir to valganciclovir for the primary efficacy endpoint of confirmed CMV viremia clearance at week 8.

## METHODS

### Study Design and Patients

This was a phase 3, randomized, multicenter, double-blind, double-dummy, active-controlled study in HCT recipients. Eligibility criteria included age 16 years or older, life expectancy of 8 weeks or more, and first documented asymptomatic CMV viremia infection (primary or reactivation) following HCT with a plasma CMV DNA load of 910 IU/mL to 91 000 IU/mL, inclusive, 2 consecutive assessments, separated by at least 1 day (determined by a local or central specialty laboratory) and without CMV tissue-invasive disease (investigator assessed). “Asymptomatic CMV infection” was defined as absence of tissue-invasive CMV disease as diagnosed by the investigator [18]. Viral load criteria were expanded after study commencement to include patients who met high-risk CMV criteria with a plasma CMV DNA load of 455 IU/mL to 910 IU/mL, inclusive (Supplementary Methods). Patients were required to have an absolute neutrophil count (ANC) of at least 1000/mm^3^, hemoglobin of at least 8 g/dL, and platelet count of at least 25 000/mm^3^ at enrollment. Patients who achieved the eligibility threshold after transfusion were eligible.

Exclusion criteria included tissue-invasive disease (investigator-assessed); confirmed CMV genotypically resistant to ganciclovir, valganciclovir, foscarnet, or cidofovir; or recurrent CMV viremia (plasma CMV DNA concentration greater than or equal to the lower limit of quantification [LLOQ] in 2 consecutive samples ≥5 days apart, after being unquantifiable [<LLOQ] for ≥5 days in 2 consecutive samples, as determined by COBAS AmpliPrep/COBAS TaqMan CMV Test, Roche Diagnostics). Patients were ineligible if they had received ganciclovir, valganciclovir, foscarnet, or letermovir for the current CMV viremia for more than 72 hours (and/or an investigational agent with known anti-CMV activity ≤30 days before study treatment initiation), a CMV vaccine, or were currently receiving leflunomide or artesunate.

**Figure 1.**
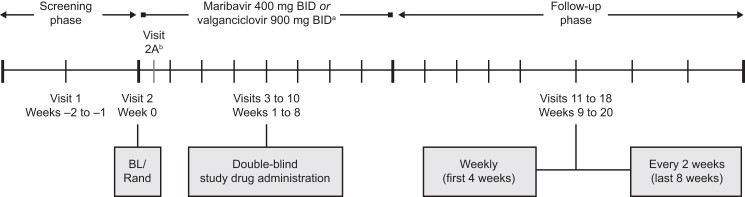
Study design. Abbreviations: BID, twice daily; BL, baseline; Rand, randomization. ^a^Unless dose adjustment was required for renal impairment. ^b^Visit 2A was required only for patients receiving tacrolimus, cyclosporine, everolimus, or sirolimus at baseline.

The study was conducted at 97 centers across North America, Europe, and Asia-Pacific. Eligible patients were randomized through interactive response technology 1:1 to receive maribavir (400 mg orally BID) or valganciclovir (orally; 900 mg BID, 450 mg BID, or 450 mg once daily, based on patients’ creatinine clearance) for 8 weeks plus 12 weeks of follow-up (Figure 1). If patients developed neutropenia (ANC <1000/mm^3^), the valganciclovir dose could be reduced to 450 mg BID or interrupted and resumed at 450 mg or 900 mg BID. Randomization was stratified by screening plasma (or equivalent whole blood) CMV DNA concentration (high, ≥9100 IU/mL to ≤91 000 IU/mL; low, ≥910 IU/mL to <9100 IU/mL; very low, ≥455 IU/mL to <910 IU/mL) and presence or absence of acute graft-versus-host disease (GVHD) [19, 20], which are associated with the risk of CMV disease and increased mortality [21, 22]. Blinding was maintained until database lock after the last patient's final visit. The Supplementary Methods presents protocol amendments implemented after trial commencement.

The study was conducted in accordance with the International Conference on Harmonization guidelines on Good Clinical Practice and the principles of the Declaration of Helsinki. Institutional review boards/independent ethics committees at each site approved the study. An independent data monitoring committee reviewed all study data at periodic intervals. All patients/legal guardians provided written informed consent.

### Endpoints and Assessments

The primary endpoint was confirmed CMV viremia clearance at the end of week 8 after receiving exclusively study-assigned treatment through week 8, regardless of early study-assigned treatment discontinuation before week 8. Confirmed CMV viremia clearance was defined as plasma CMV DNA concentration below the LLOQ (<137 IU/mL) when assessed by COBAS AmpliPrep/COBAS TaqMan CMV Test at a central specialty laboratory in 2 consecutive postbaseline samples, separated by at least 5 days.

The key secondary endpoint was a composite of the primary endpoint with no clinical findings of CMV tissue-invasive disease [18] at the end of week 8, followed by maintenance of this treatment effect through week 16 (8 weeks beyond the treatment phase) after receiving exclusively study-assigned treatment. Efficacy was also evaluated in prespecified subgroups.

Safety endpoints included treatment-emergent adverse events (TEAEs) and treatment-emergent serious adverse events (TESAEs). Additional endpoints and study assessment details are provided in the Supplementary Methods.

### Statistical Analysis

To declare noninferiority of maribavir to valganciclovir for the primary endpoint with greater than 90% power, 550 patients (275 patients per treatment arm) were required to be enrolled. The Supplementary Methods provides additional details on sample size calculation. Primary and secondary endpoint analyses were conducted in all randomized patients who received at least 1 dose of study-assigned treatment (Modified Randomized Population). The difference between treatment arms in the proportion of patients achieving the primary endpoint was obtained using the Cochran–Mantel–Haenszel weighted average across strata of baseline plasma CMV DNA concentration (high, low, very low) and baseline acute GVHD (present, absent). The 95% confidence intervals (CIs) of the weighted average of difference across strata were calculated using normal approximation. The study hypothesized noninferiority of maribavir to valganciclovir for the primary efficacy endpoint. If the 95% CI lower limit of the weighted average of difference was greater than −7.0%, maribavir was considered as efficacious as valganciclovir. Noninferiority of the secondary endpoint was to be tested using the same method, only after noninferiority of the primary efficacy endpoint was established. Hypothesis testing of the primary and key secondary efficacy endpoints was adjusted for multiple comparisons using a gatekeeping procedure to control the family-wise type 1 error rate (2-sided α = 5% level). For other efficacy analyses, *P* values were nominal; no adjustment was made for multiple comparisons.

Prespecified subgroup analyses were performed for the primary and key secondary efficacy endpoints using similar methods for the primary endpoint, including any applicable stratification factors. There was no control of multiple testing for the subgroup analyses.

Safety data were analyzed descriptively in all patients who received at least 1 dose of study-assigned treatment (Safety Population).

## RESULTS

### Patients

**Figure 2.**
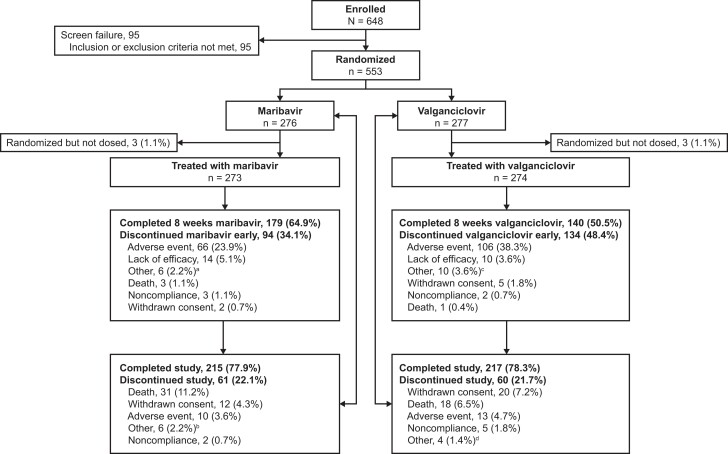
Patient disposition at enrollment, randomization, and follow-up. Percentages are based on the number of patients randomized per arm as the denominator (maribavir, n = 276; valganciclovir, n = 277). In the maribavir arm, the reasons for the 3 patients who were randomized but not dosed were withdrawal from the study (n = 2) and adverse event due to neutropenia (n = 1). In the valganciclovir arm, the reasons for the 3 patients who were randomized but not dosed were withdrawal from the study (n = 2) and other (1 patient was randomized by interactive response technology by error). Abbreviations: CMV, cytomegalovirus; COVID-19, coronavirus disease 2019; N, number of patients enrolled; n, number of patients through trial; PCR, polymerase chain reaction. ^a^Other reasons for treatment discontinuation in the maribavir arm included patient decision (n = 1), poor condition of the patient (n = 1), negative CMV virology (n = 1), difficulties in swallowing (n = 1), investigator decision (n = 1), and starting another treatment for BK virus with a prohibited medication (n = 1). ^b^Other reasons for study discontinuation in the maribavir arm included patient request (n = 2), COVID-19 pandemic (n = 1), misinterpretation of the protocol by site staff (n = 1), poor condition of the patient (n = 1), and COVID-19 infection (n = 1). ^c^Other reasons for treatment discontinuation in the valganciclovir arm included investigator decision due to negative CMV PCR or favorable CMV levels (n = 4), unspecified investigator decision (n = 1), investigator decision due to patient hospitalization (n = 1), issue with study drug dispensing/assignment (n = 1), persistent cytopenias (n = 1), patient decision (n = 1), and investigator decision due to treatment being no longer clinically necessary (n = 1). ^d^Other reasons for study discontinuation in the valganciclovir arm included reason unknown (n = 1), screen failure (n = 1), patient decision (n = 1), and physician decision due to a third reactivation of CMV (n = 1).

The study was conducted between April 2017 and July 2022; of 553 randomized patients (maribavir, 276; valganciclovir, 277), 3 patients per treatment arm did not receive the study drug (Figure 2). Demographics and baseline characteristics for the remaining patients (Modified Randomized Population) were generally balanced between the 2 arms (Table 1). All except 1 patient in each treatment arm received allogeneic HCT. Of the randomized patients, 215 (77.9%) and 217 (78.3%) who received maribavir and valganciclovir, respectively, completed the study (Figure 2). The median time on study was 141 days in each treatment arm (range: maribavir, 1–307 days; valganciclovir, 1–351 days). Adverse events were the most frequent reason for early treatment discontinuation (Figure 2).

**Table 1.** Baseline Demographics and Characteristics (Modified Randomized Population)

Characteristic	Maribavir (n = 273)	Valganciclovir (n = 274)
Mean (SD) age, y	53.2 (13.87)	51.7 (15.24)
Age category, n (%)		
<18 y	1 (0.4)	3 (1.1)
18–44 y	57 (20.9)	78 (28.5)
45–64 y	157 (57.5)	125 (45.6)
≥65 y	58 (21.2)	68 (24.8)
Sex, n (%)		
Male	148 (54.2)	165 (60.2)
Female	125 (45.8)	109 (39.8)
Race, n (%)		
White	218 (79.9)	198 (72.3)
Black or African-American	10 (3.7)	9 (3.3)
Native Hawaiian or other Pacific Island	0	3 (1.1)
Asian	36 (13.2)	39 (14.2)
American Indian or Alaska Native	0	1 (0.4)
Other	7 (2.6)	20 (7.3)
Missing	2 (0.7)	4 (1.5)
Enrolling region, n (%)		
Asia	48 (17.6)	42 (15.3)
Europe	159 (58.2)	174 (63.5)
North America	66 (24.2)	58 (21.2)
Median plasma CMV DNA by central laboratory at baseline, IU/mL (Q1, Q3)	2042.0 (776.0, 6629.5)	2076.0 (748.0, 5742.0)
Plasma CMV DNA category by central laboratory, n (%)		
Very low (≥455 to <910 IU/mL)	78 (28.6)	78 (28.5)
Low (≥910 to <9100 IU/mL)	145 (53.1)	147 (53.6)
High (≥9100 IU/mL)	49 (17.9)	48 (17.5)
Missing	1 (0.4)	1 (0.4)
Acute GVHD status, n (%)		
Absence	221 (81.0)	223 (81.4)
Presence	52 (19.0)	51 (18.6)
Chronic GVHD status, n (%)		
Absence	267 (97.8)	259 (94.5)
Presence	6 (2.2)	15 (5.5)
Type of preparative condition regimen, n (%)		
Myeloablative	98 (35.9)	102 (37.2)
Non-myeloablative	41 (15.0)	60 (21.9)
Reduced-intensity conditioning regimen	129 (47.3)	105 (38.3)
Not applicable	3 (1.1)	4 (1.5)
Missing	2 (0.7)	3 (1.1)
CMV serostatus, n (%)		
Donor +/recipient +	138 (50.5)	155 (56.6)
Donor −/recipient +	98 (35.9)	83 (30.3)
Donor +/recipient −	20 (7.3)	22 (8.0)
Donor −/recipient −	10 (3.7)	10 (3.6)
Missing	7 (2.6)	4 (1.5)
Use of T-cell depletion therapy, n (%)		
Yes	127 (46.5)	141 (51.5)
No	146 (53.5)	133 (48.5)
Type of T-cell depletion agent, n (%)		
Alemtuzumab	15 (5.5)	10 (3.6)
Anti-thymocyte immunoglobulin	89 (32.6)	109 (39.8)
Ex vivo T-cell depletion	23 (8.4)	22 (8.0)
Patients with prior HCT transplant, n (%)		
0 transplants	230 (84.2)	250 (91.2)
1 transplants	37 (13.6)	22 (8.0)
2 transplants	6 (2.2)	2 (0.7)
≥3 transplants	0	0
Type of transplant, n (%)		
Autologous	1 (0.4)	1 (0.4)
Allogeneic	272 (99.6)	273 (99.6)
Donor type for allogeneic HCT,^a^ n (%)		
HLA-identical sibling	42 (15.4)	38 (13.9)
HLA-matched other relative	53 (19.5)	54 (19.8)
HLA-mismatched relative	62 (22.8)	55 (20.1)
Syngeneic	0	1 (0.4)
Unrelated donor	115 (42.3)	125 (45.8)
Reason for current transplant, n (%)		
Leukemia (acute myeloid)	97 (35.5)	111 (40.5)
Leukemia (chronic myeloid)	8 (2.9)	9 (3.3)
Leukemia (acute lymphoblastic)	30 (11.0)	32 (11.7)
Lymphoma (non-Hodgkin's)	20 (7.3)	14 (5.1)
Myelodysplastic syndrome	41 (15.0)	40 (14.6)
Other myeloid malignancy	6 (2.2)	10 (3.6)
Other	71 (26.0)	58 (21.2)
History of CMV prophylaxis, n (%)		
Yes	26 (9.5)	22 (8.0)
No	247 (90.5)	252 (92.0)
Median (min–max) time from current HCT to the first dose of study treatment, d	48 (16–370)	49 (18–328)

Abbreviations: CMV, cytomegalovirus; GVHD, graft-versus-host disease; HCT, hematopoietic cell transplant; HLA, human leukocyte antigen; max, maximum; min, minimum; Q, quartile; SD, standard deviation; +, positive; −, negative.

^a^Percentages are based on the number of patients within the category indicated.

### Efficacy

#### Primary Endpoint

**Figure 3.**
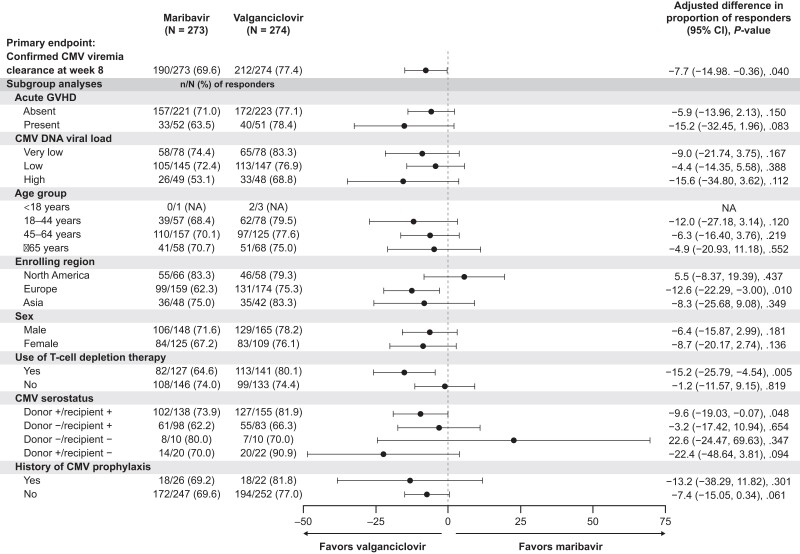
Confirmed CMV viremia clearance at week 8 (primary endpoint) and prespecified subgroup analyses of confirmed CMV viremia clearance at week 8 by treatment arm (Modified Randomized Population). The Cochran–Mantel–Haenszel weighted average approach was used for the adjusted difference in proportion (maribavir−valganciclovir) and the corresponding 95% CI, adjusting for acute GVHD and baseline plasma CMV DNA concentration. Abbreviations: CI, confidence interval; CMV, cytomegalovirus; GVHD, graft-versus-host disease; N, number of patients in a treatment arm; n, number of responders; NA, not applicable.

At week 8, 190 of 273 (69.6%) and 212 of 274 (77.4%) patients in the maribavir and valganciclovir arms, respectively, achieved confirmed CMV viremia clearance (adjusted difference: −7.7%; 95% CI: −14.98, −.36) (Figure 3). The criterion for noninferiority of maribavir to valganciclovir for the primary endpoint was not met because the lower limit of the 95% CI of treatment difference was below −7.0%. Results for most prespecified subgroup analyses were numerically consistent with those for the primary analysis (Figure 3). For patients with high viral load, acute GVHD, and those who had undergone T-cell depletion at baseline, the treatment effect of maribavir was below the group average. Reasons for not achieving the primary endpoint in the maribavir and valganciclovir arms, respectively, were as follows: administration of non-study anti-CMV drugs during the treatment phase (13.6% and 9.9%), documented failure to achieve confirmed CMV viremia clearance (7.7% and 2.2%), and missing CMV DNA to assess response at week 8 (9.2% and 10.6%). These reasons were mutually exclusive—that is, patients with “documented failure to achieve confirmed CMV viremia clearance” did not receive non-study anti-CMV drugs before week 8 and did not achieve CMV viremia clearance during the treatment phase, or they did achieve CMV viremia clearance but subsequently had CMV viremia recurrence by week 8.

#### Key Secondary Endpoint

**Figure 4.**
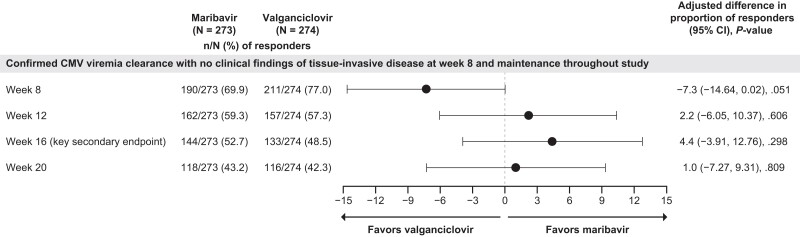
Maintenance of confirmed CMV viremia clearance with no clinical findings of tissue-invasive disease achieved at week 8 through weeks 12, 16 (key secondary endpoint), and 20 (Modified Randomized Population). Abbreviations: CI, confidence interval; CMV, cytomegalovirus; N, number of patients in a treatment arm; n, number of responders.

A similar proportion of patients treated with maribavir and valganciclovir achieved CMV viremia clearance with no clinical findings of CMV tissue-invasive disease at week 8, with maintenance through week 16 (52.7% vs 48.5%; adjusted difference: 4.4; 95% CI: −3.91, 12.76) (Figure 4). Prespecified subgroup analyses were generally consistent with this finding; however, the treatment effect of maribavir was below the group average in patients with high viral load, acute GVHD, and those who had previously undergone T-cell depletion (Supplementary Figure 1).

#### Other Secondary Endpoints

Maribavir and valganciclovir were comparable for the maintenance of CMV viremia clearance with no clinical findings of CMV tissue-invasive disease at post-treatment evaluations (weeks 12 and 20) (Figure 4).

Of patients who achieved confirmed CMV viremia clearance at any time (ie, between weeks 1 and 20) in the maribavir (n = 226) and valganciclovir (n = 236) arms, 19.0% and 22.5%, respectively, had CMV viremia recurrence. Recurrence during the treatment phase of the study (ie, weeks 1–8) was more frequent with maribavir (7.1%) than valganciclovir (2.5%). While still receiving the study drug, 14 patients in the maribavir arm and none in the valganciclovir arm had CMV viremia recurrence. Recurrence after week 8 was more frequent with valganciclovir (19.9%) than maribavir (11.9%).

#### Exploratory and Post Hoc Evaluations

**Figure 5.**
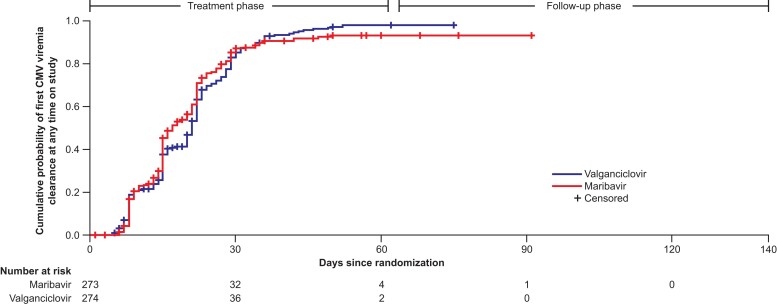
Cumulative probability of first CMV viremia clearance at any time on study by treatment arm (Modified Randomized Population). Abbreviation: CMV, cytomegalovirus.

**Figure 6.**
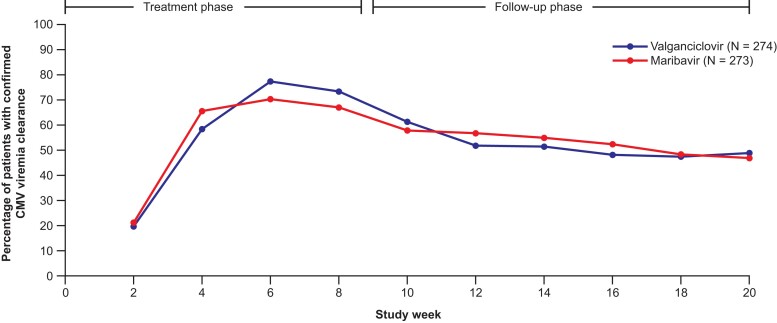
Percentage of patients with confirmed CMV viremia clearance by study week and treatment arm (Modified Randomized Population; post hoc analysis). A post hoc analysis of patients with confirmed CMV viremia clearance by study week for the 2 treatment arms was performed by calculating the proportion of patients with CMV DNA below the lower limit of quantification at that visit and consecutive prior visits spanning 5 days (ie, confirmed viremia clearance by central laboratory) for each treatment arm. Abbreviation: CMV, cytomegalovirus.

Overall, 82.8% and 86.1% of patients treated with maribavir and valganciclovir, respectively, achieved confirmed CMV viremia clearance at any time within the first 8 weeks of the study (adjusted difference: −3.2; 95% CI: −9.19, 2.72; *P* = .287). Of these, some patients may have had recurrence by week 8, thus explaining the higher proportion of patients who achieved CMV viremia clearance compared with the primary endpoint result. Kaplan–Meier median (95% CI) time to first confirmed CMV viremia clearance at any time on study was 17.0 (15.0, 20.0) days with maribavir and 21.0 (20.0, 22.0) days with valganciclovir (Figure 5). A post hoc analysis of the percentage of patients with confirmed CMV viremia clearance over time by week for each treatment arm is presented in Figure 6.

Treatment-emergent resistance occurred in 8.8% and 2.9% of patients in the maribavir and valganciclovir arms, respectively; 21 patients in the maribavir arm and 4 in the valganciclovir arm who developed treatment-emergent mutations did not achieve the primary endpoint. These results can be described as follows: in the maribavir arm, 19 patients developed resistance to only maribavir, 1 patient developed resistance to only valganciclovir, and 1 developed resistance to both maribavir and valganciclovir; in the valganciclovir arm, 3 patients developed resistance to only valganciclovir and 1 developed resistance to both maribavir and valganciclovir. Of the 14 patients with CMV viremia recurrence on maribavir treatment, 12 (85.7%) had treatment-emergent resistance mutations. No patients treated with valganciclovir had a recurrence on treatment. A list of treatment-emergent mutations is provided in Supplementary Tables 2 and 3.

With maribavir and valganciclovir, all-cause mortality was 13.6% and 10.6% (Supplementary Results), respectively, and tissue-invasive disease developed in 3.3% and 3.6% of patients, respectively.

### Safety

The median (range) duration of exposure was 56 (1–62) days with maribavir and 54 (2–63) days with valganciclovir. In both treatment arms, 98.2% of patients had at least 1 TEAE during the 8-week on-treatment phase (Table 2). Treatment-related TEAEs were more frequent with valganciclovir (61.3% patients) than maribavir (54.2% patients). A smaller proportion of patients discontinued study treatment due to a TEAE with maribavir (27.8%) than with valganciclovir (41.2%). Patients most commonly discontinued treatment due to TEAEs of neutropenia (maribavir, 4.0%; valganciclovir, 17.5%).

**Table 2.** Treatment-Emergent Adverse Events in More Than 5% of Patients During the On-Treatment Phase by System Organ Class, Preferred Term, and Treatment Arm (Safety Population)

System Organ Class Preferred Term	Maribavir (n = 273)	Valganciclovir (n = 274)
Any TEAE	268 (98.2)	269 (98.2)
Blood and lymphatic system disorders	125 (45.8)	187 (68.2)
Anemia	63 (23.1)	50 (18.2)
Leukopenia	7 (2.6)	27 (9.9)
Neutropenia	44 (16.1)	145 (52.9)
Thrombocytopenia	31 (11.4)	63 (23.0)
Gastrointestinal disorders	162 (59.3)	140 (51.1)
Abdominal pain	14 (5.1)	19 (6.9)
Constipation	16 (5.9)	10 (3.6)
Diarrhea	53 (19.4)	47 (17.2)
Nausea	75 (27.5)	64 (23.4)
Vomiting	57 (20.9)	47 (17.2)
General disorders and administration-site conditions	96 (35.2)	95 (34.7)
Asthenia	13 (4.8)	19 (6.9)
Fatigue	13 (4.8)	19 (6.9)
Edema peripheral	27 (9.9)	26 (9.5)
Pyrexia	30 (11.0)	34 (12.4)
Immune system disorders	73 (26.7)	58 (21.2)
Acute GVHD in intestine	18 (6.6)	11 (4.0)
Acute GVHD in skin	47 (17.2)	32 (11.7)
Investigations	95 (34.8)	106 (38.7)
Blood creatinine increased	18 (6.6)	12 (4.4)
Neutrophil count decreased	13 (4.8)	29 (10.6)
Platelet count decreased	17 (6.2)	16 (5.8)
White blood cell count decreased	3 (1.1)	14 (5.1)
Metabolism and nutrition disorders	93 (34.1)	82 (29.9)
Decreased appetite	18 (6.6)	16 (5.8)
Hypokalemia	23 (8.4)	22 (8.0)
Nervous system disorders	119 (43.6)	70 (25.5)
Dysgeusia	47 (17.2)	16 (5.8)
Headache	30 (11.0)	17 (6.2)
Taste disorder	23 (8.4)	6 (2.2)
Tremor	11 (4.0)	15 (5.5)
Renal and urinary disorders	61 (22.3)	58 (21.2)
Renal impairments	25 (9.2)	15 (5.5)
Respiratory, thoracic, and mediastinal disorders	54 (19.8)	66 (24.1)
Cough	21 (7.7)	26 (9.5)
Dyspnea	5 (1.8)	14 (5.1)
Skin and subcutaneous tissue disorders	61 (22.3)	64 (23.4)
Pruritus	10 (3.7)	17 (6.2)
Vascular disorders	34 (12.5)	30 (10.9)
Hypertension	14 (5.1)	17 (6.2)

Data are presented as n (%). Percentages are based on the number of patients in the Safety Population within each column. Patients were counted once per System Organ Class and once per Preferred Term, per treatment. The on-treatment phase started at the time of study treatment initiation through 7 days after the last dose of study treatment, or until the non-study CMV treatment initiation, whichever was earlier. TEAEs were defined as any adverse event occurring during the on-treatment phase. Adverse events were coded using MedDRA, version 23.0.

Abbreviations: CMV, cytomegalovirus; GVHD, graft-versus-host disease; MedDRA, Medical Dictionary for Regulatory Activities; TEAE, treatment-emergent adverse event.

Nausea was the most frequently reported TEAE with maribavir (27.5%; valganciclovir, 23.4%). Dysgeusia (abnormal taste) was more frequently reported as a TEAE with maribavir (17.2%) than with valganciclovir (5.8%) and was the most frequently reported treatment-related TEAE with maribavir (13.9%; valganciclovir, 4.4%). Dysgeusia usually resolved on treatment or within 11 days after treatment discontinuation. Dysgeusia with maribavir led to treatment discontinuation for 5 (1.8%) patients.

Neutropenia as an adverse event of special interest (ie, comprised events of agranulocytosis, febrile neutropenia, neutropenia, and decreased neutrophil count) occurred in more patients treated with valganciclovir (63.5%) than with maribavir (21.2%). More patients treated with valganciclovir than maribavir experienced grade 3 (ANC <1000/mm^3^) or 4 (ANC <500/mm^3^) neutropenia (50.0% and 16.1%, respectively). Compared with maribavir, neutropenia was associated with greater use of granulocyte-colony stimulating factor (GCSF) with valganciclovir, both during study treatment (valganciclovir, 15.3% of patients; maribavir, 6.2% of patients) and after study treatment discontinuation (valganciclovir, 15.0% of patients; maribavir, 9.2% of patients), and also led to hospitalization more frequently in the valganciclovir arm (6.2% of patients; maribavir, 2.2% of patients).

New-onset GVHD was reported on-treatment for 22.7% and 18.2% of maribavir- and valganciclovir-treated patients, respectively. Of patients with GVHD at baseline, 44 of 57 (77.2%) and 45 of 63 (71.4%) patients treated with maribavir and valganciclovir, respectively, experienced no new or worsened GVHD on-treatment. The incidence of TESAEs was similar between maribavir (32.2%) and valganciclovir (34.7%). During the on-treatment phase, TESAEs led to 18 (6.6%) and 12 (4.4%) deaths in the maribavir and valganciclovir arms, respectively (Supplementary Table 1). No deaths were attributed to the study drug.

## DISCUSSION

Despite the results of a smaller open-label phase 2 trial that previously compared the efficacy of maribavir and valganciclovir for asymptomatic CMV viremia following HCT and solid-organ transplant [17], this larger double-blind phase 3 trial in HCT recipients did not demonstrate noninferiority of maribavir to valganciclovir for the primary endpoint of confirmed CMV viremia clearance at week 8. Nonetheless, during the post-treatment phase, a similar proportion of patients maintained CMV viremia clearance without tissue-invasive disease in both treatment arms. Safety findings for maribavir in this study were consistent with prior studies [6, 17, 23]. Notably, maribavir was associated with a lower incidence of neutropenia leading to treatment discontinuation, GCSF use, and hospitalizations than valganciclovir. The TESAEs associated with death were similar and low with both treatments. Thus, the authors pose that outcomes with maribavir in the study are clinically meaningful for the transplant patient population given that (1) viral load is an appropriate surrogate endpoint for CMV trials in transplant recipients [24], (2) efficacy of anti-CMV treatment versus placebo is long established [25], (3) strategies with comparable efficacy and safety that improve convenience to the patient and healthcare system (eg, oral valganciclovir vs intravenous formulation of ganciclovir) are beneficial [26], and (4) there remains an unmet need for an effective anti-CMV therapy with fewer treatment-limiting toxicities.

The study not meeting its primary endpoint is partially attributed to the development of resistance to maribavir during the 8-week treatment phase. Compared with valganciclovir, more patients in the maribavir arm developed treatment-emergent mutations during the treatment phase and did not achieve the primary endpoint. Notably, CMV strains resistant to maribavir usually remain susceptible to valganciclovir/ganciclovir, although some mutations associated with resistance to maribavir confer cross-resistance to valganciclovir/ganciclovir and require treatment with foscarnet [27, 28]. Furthermore, the noninferiority margin of the primary endpoint was conservative and smaller than the margins commonly used in other treatment studies for this complex population [26, 29–32]. The study also excluded patients with an ANC of less than 1000/mm^3^ and platelet count of less than 25 000/mm^3^ (who were less likely to tolerate 8 weeks of valganciclovir treatment from a hematologic perspective), and anti-CMV therapy was administered for a fixed duration of 8 weeks, with valganciclovir dose adjustments permitted for patients who developed neutropenia or had renal dysfunction. In contrast, in clinical practice, therapy is typically given until CMV viremia clearance (which may occur earlier or later than 8 weeks) [33], and valganciclovir may be discontinued in patients unable to tolerate it [7, 8]. Other baseline differences between the study arms may also have contributed to an imbalance in later complications (eg, the development of GVHD necessitating additional immunosuppression) [34].

Maribavir was numerically less effective than valganciclovir for patients with high viral load, acute GVHD, and T-cell depletion; however, inferences about the statistical significance of these findings should not be made as the *P* values were nominal and lacked multiplicity controls. In clinical practice, patients with GVHD and/or T-cell depletion may require longer pre-emptive treatment, which introduces the risk of developing resistance in the setting of active CMV replication. In highly immunocompromised patients, pre-emptive treatment is initiated at low viral loads to prevent the emergence of resistance [11, 35].

Beyond the week 8 time point chosen for the primary endpoint in this trial, a post hoc analysis showed that the difference in the proportion of patients achieving confirmed viremia clearance with maribavir or valganciclovir varied over time. At all post-treatment endpoints, maribavir and valganciclovir demonstrated comparable CMV viremia clearance rates with no clinical findings of tissue-invasive disease. Importantly, only 3.0% and 3.6% of patients treated with maribavir and valganciclovir, respectively, developed CMV tissue-invasive disease, confirming that both treatments are effective for the prevention of CMV tissue-invasive disease in the setting of proper virologic monitoring.

As expected from prior studies, dysgeusia occurred more often with maribavir than valganciclovir, which rarely led to treatment discontinuation [6, 36]. The observation of fewer discontinuations attributed to neutropenia in the maribavir arm of this study, along with substantially lower overall rates of neutropenia (including grade 3 or 4) compared with valganciclovir, merits discussion. Management of neutropenia is a serious challenge for HCT recipients as it carries the risk of secondary bacterial/fungal infections and neutropenic fever [17, 37], thus increasing hospitalization risk. Neutropenia may also lead to immunosuppressant dose reductions, increasing the risk of GVHD. Additionally, the need for growth factors to manage neutropenia can increase treatment costs; indeed, in this study, fewer patients treated with maribavir than with valganciclovir required GCSF.

One of the limitations of this study is the nonavailability of data on immunosuppressive therapy for active GVHD post-treatment. Therapy for active GVHD during the post-treatment phase likely contributed to the lower percentage of patients in both treatment arms with maintenance of CMV viremia clearance with no clinical findings of CMV-invasive tissue disease at week 16 (ie, after treatment) compared with week 8 (ie, on treatment). Other limitations are inherent to the design of the study; for example, in real-world practice, a fixed duration of antiviral treatment for CMV infection is not necessarily for 8 weeks.

In conclusion, although noninferiority of maribavir to valganciclovir was not met based on the predefined noninferiority margin of 7.0% at week 8, the anti-CMV activity of maribavir was comparable at other time points (weeks 12, 16, and 20) in patients with first asymptomatic CMV infection post-HCT. Other clinical outcomes were comparable for maribavir and valganciclovir. Safety outcomes of maribavir were consistent with prior studies; when compared with valganciclovir, maribavir treatment was associated with fewer treatment discontinuations, GCSF use, and hospitalizations due to adverse events of neutropenia.

## Supplementary Data

Supplementary materials are available at *Clinical Infectious Diseases* online. Consisting of data provided by the authors to benefit the reader, the posted materials are not copyedited and are the sole responsibility of the authors, so questions or comments should be addressed to the corresponding author.

## Supplementary Material

ciad709_Supplementary_Data

## Notes

***Author Contributions.*** All authors were involved in drafting the article and revising it critically for important intellectual content, and all authors approved the final version to be published. Study conception and design: G. A. P., M. F., R. A. M., and J. W. Acquisition of data: G. A. P., R. K. A., C. C., R. F. D., S. H., J. M., K. S. P., C. S., J.-A. H. Y., and D. J. W. Analysis and interpretation of data: G. A. P., R. K. A., C. C., R. F. D., S. H., J. M., K. S. P., C. S., J.-A. H. Y., M. F., R. A. M., J. W., and D. J. W.

***Acknowledgments.*** The authors thank all the patients who participated in this trial as well as their families. Under the direction of the authors, Ashvanti Valji, PhD, of Caudex, UK, a division of IPG Health Medical Communications, provided writing assistance, and Mary Casey, PhD, of Caudex, UK, provided editorial assistance. Caudex was funded by Takeda Development Center Americas, Inc, Lexington, Massachusetts, USA, for medical writing and editorial services.

***Financial support***. This work was supported by Takeda Development Center Americas, Inc, Lexington, Massachusetts, USA.
