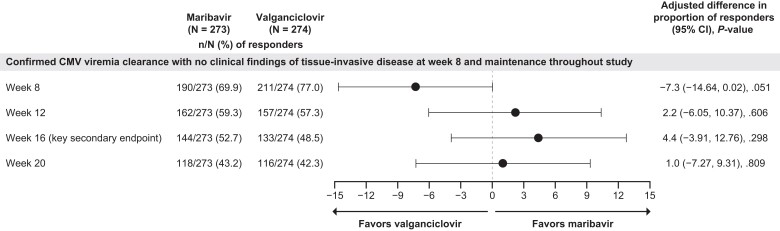# Correction to: Treatment for First Cytomegalovirus Infection Post–Hematopoietic Cell Transplant in the AURORA Trial: A Multicenter, Double-Blind, Randomized, Phase 3 Trial Comparing Maribavir With Valganciclovir

**DOI:** 10.1093/cid/ciae356

**Published:** 2024-08-02

Genovefa A. Papanicolaou, Robin K. Avery, Catherine Cordonnier, Rafael F. Duarte, Shariq Haider, Johan Maertens, Karl S. Peggs, Carlos Solano, Jo-Anne H. Young, Martha Fournier, Rose Ann Murray, Jingyang Wu, and Drew J. Winston; for the AURORA Trial Investigators

In the originally published version of this article [Papanicolaou GA, Avery RK, Cordonnier C, et al. Treatment for First Cytomegalovirus Infection Post–Hematopoietic Cell Transplant in the AURORA Trial: A Multicenter, Double-Blind, Randomized, Phase 3 Trial Comparing Maribavir With Valganciclovir. *Clin Infect Dis* 2024. https://doi.org/10.1093/cid/ciad709], there was an error in Figure 4. The 95% confidence interval for the adjusted difference in proportion of responders at Week 8 shown in the forest plot should have crossed 0 in line with the values reported in the last column of the table (−14.64, 0.02). The figure has been updated in the article.

**Figure:**