# Supplementary material for: Treatment for First Cytomegalovirus Infection Post–Hematopoietic Cell Transplant in the AURORA Trial: A Multicenter, Double-Blind, Randomized, Phase 3 Trial Comparing Maribavir With Valganciclovir
Source: Clin Infect Dis. 2023 Nov 30;78(3):562–72. doi: 10.1093/cid/ciad709 (PMC10954327; doi:10.1093/cid/ciad709)
Supplement: ciad709_Supplementary_Data [file ciad709_supplementary_data.pdf]

Supplementary Materials

Treatment for First Cytomegalovirus Infection Post-Hematopoietic Cell Transplant in the AURORA trial: A Multicenter, Double-Blind, Randomized, Phase 3 Trial Comparing Maribavir With Valganciclovir

Papanicolaou GA, et al.

Contents

Investigators 2
Supplementary Methods 4
Study Design and Patients 4
Protocol Amendments 4
Additional Endpoints 5
Trial Monitoring and Follow-up 5
Statistical Analyses 6
Supplementary Results 7
All-cause mortality 7
Supplementary Table 1. Treatment-Emergent Adverse Events Leading to Death During the On-Treatment Phase by System Organ Class, Preferred Term, and Treatment Arm (Safety Population) 8
Supplementary Table 2. Identified Treatment-Emergent[a] Known or Suspected RASs to Conventional Anti-CMV Therapies[b] 10
Supplementary Table 3. Identified Treatment-Emergent[a] Known or Suspected RASs to Maribavir[b] 11
Supplementary Figure 1. Subgroup analysis of confirmed CMV viremia clearance with no clinical findings of tissue-invasive disease at week 8 with maintenance of this treatment effect through week 16, by treatment arm. 12

Investigators

The following investigators participated in the AURORA study: **Australia:** Deepak Singhal, Royal Adelaide Hospital, Adelaide; Joe Sasadeusz, Royal Melbourne Hospital, Melbourne; **Belgium**: Johan Maertans, University Hospitals Leuven, Leuven, Aspasia Georgala, Institut Jules Bordet, Brussels; Dominik Selleslag, Hospital AZ Sint Jan, Bruges; Anke Verlinden, Antwerp University Hospital, Edegem; Tessa Kerre, Ghent University and Ghent University Hospital, Ghent; Ann De Becker, Universitair Ziekenhuis Brussels; **Canada**: Shariq Haider, Hamilton Health Sciences Corporation, Hamilton; Alissa Wright, Vancouver General Hospital, Vancouver; **China**: Depei Wu, The First Affiliated Hospital of Soochow University, Suzhou; **Croatia**: Radovan Vrhovac, University Hospital Center Zagreb, Zagreb; **France**: Catherine Cordonnier, Henri Mondor Hôpital, Créteil; Ana Berceanu, University Hospital of Besançon, Besançon; Sylvie Francois, Maladies du Sang, Angers; David Michonneau, Hôpital Saint Louis, Paris; Anne Huynh, Cancer Universitary Institute, Toulouse; **Germany**: Wolfgang Bethge, Universitätsklinikum Tübingen, Tübingen; Martin Kaufmann, Robert Bosch Hospital, Stuttgart; Matthias Stelljes, Universitätsklinikum Münster, Münster; Georg-Nikolaus Franke, Universitatsklinikum Leipzig, Leipzig; Timo Schmitt, Universitätsmedizin der Johannes Gutenberg-Universität Mainz, Mainz; Lutz Müller, Martin Luther Universitat Halle Wittenberg, Halle; Manfred Ahlgrimm, Universität des Saarlandes, Homburg; Judith Niederland, Helios Klinikum Berlin-Buch, Berlin; **Greece**: Panagiotis Tsirigotis, Attikon University General Hospital, Athens; **Israel**: Ron Ram, Tel Aviv Sourasky Medical Center PPDS, Tel Aviv; Noga Shemtov, Sheba Medical Center – PPDS, Ramat Gan; Tsila Rosenvald-Zuckerman, Rambam Medical Center – PPDS, Haifa; **Italy**: Ilaria Cutini, Azienda Ospedaliera Universitaria Careggi, Florence; Alessandro Busca, Azienda Ospedaliera Città della Salute e della Scienza di Torino, Turin; Francesco Onida, Fondazione IRCCS Cà Granda Ospedale Maggiore Policlinico, Milan; Cristina Tecchio, Azienda Ospedaliera Universitaria Integrata Di Verona, Verona; **New Zealand**: Peter Browett, Auckland City Hospital, Auckland; **Republic of Korea**: Young Rok Do, Keimyung University Dongsan Hospital, Daegu; Sung Hyun Kim, Dong-A University Hospital, Busan; **Singapore**: Aloysius Ho, Singapore General Hospital, Singapore; Liang Piu Koh, National University Hospital, Singapore; **Spain**: Maria Lourdes Vazquez Lopez, Complejo Asistencial Universitario de Salamanca – H. Clinico, Salamanca; Javier Lopez Jimenez, Hospital Universitario Ramon y Cajal, Madrid; Christelle Ferra Coll, Hospital Universitario Germans Trias I Pujol, Badalona; Rafael De la Camara, Hospital Universitario de La Princesa, Madrid; Carlos Solano, Hospital Clinico Universitario de Valencia, Valencia; Alberto Mussetti, ICO l'Hospitalet – Hospital Duran i Reynals, Barcelona; Juan Carlos Vallejo Llamas, Hospital Universitario de Donostia, San Sebastián; Pere Barba Suñol, Hospital Universitario Vall d'Hebron - PPDS, Barcelona;

Manuel Jurado Chacón, Hospital Universitario Virgen de Las Nieves, Granada; Rafael F. Duarte, Hospital Universitario Puerta de Hierro Majadahonda, Madrid; María Aranzazu Bermúdez Rodríguez, Hospital Universitario Marques de Valdecilla, Santander; **Switzerland**: Nicolas Mueller, Universitätsspital Zürich, Zürich; **Turkey**: Hakan Ozdogu, Baskent University Medical Faculty Adana Practice and Research Center, Adana; Gunhan Gurman, Ankara University Medica Faculty Hematology Department Clinical Research Area PPDS, Ankara; **United Kingdom**: Adrian Bloor, The Christie NHS Foundation Trust – PPDS, Manchester; Bhuvan Kishore, Birmingham Heartlands Hospital, Birmingham; Karl S. Peggs, University College London, London; Dragana Milojkovic, Hammersmith Hospital, London; Kim Orchard, Southampton University Hospitals NHS Trust, Southampton; Arpad Gabor Toth, Clatterbridge Cancer Centre Liverpool, Liverpool; Mickey Koh, St George's Hospital, London; **United States**: Robin K. Avery, Johns Hopkins Hospital, Baltimore; Jennifer Pisano, University of Chicago, Chicago; George Alangaden, Henry Ford Health System, Detriot; Drew J. Winston, UCLA Center for Health Sciences, Los Angeles; Genovefa Papanicolau, Memorial Sloan Kettering Cancer Center, New York; Benjamin Gewurz, Brigham and Womens Hospital, Boston; Francisco M Marty*, Dana-Farber Cancer, Brigham and Women's Hospital, Harvard Medical School, Boston; Jo-Anne H. Young, University of Minnesota, Minneapolis; Patrick Hagen, Loyola University Medical Center, Chicago; Ran Reshef, Columbia University Medical Center, New York; Sameem Abedin, The Medical College of Wisconsin, Inc., Milwaukee; Paul Shaughnessy, Texas Transplant Institute, San Antonio; Laura Gibson, UMass Memorial Medical Center, Worcester; Tsiporah Shore, Joan and Sandford I. Weill Medical College of Cornell University Clinic, New York; Carlos R. Bachier, Sarah Cannon Center for Blood Cancer, Nashville; Jean Yared, University of Maryland School of Medicine, Baltimore; Maricar Malinis, Yale University School of Medicine, Connecticut.

*****Dr. Marty is deceased.

Supplementary Methods

Study Design and Patients

High-risk Cytomegalovirus (CMV) Inclusion Criteria for Patients with Low Viral Load

Patients with plasma CMV DNA ≥455 IU/mL to <910 (or whole blood equivalent) had to meet at least one of the following criteria for high-risk CMV infection to be eligible for inclusion:

1. Human leukocyte antigen (HLA)-related (sibling) donor with ≥1 mismatch at 1 of 3 HLA-gene loci (HLA-A, -B, or -DR)
2. Haploidentical donor
3. Unrelated donor with ≥1 mismatch at 1 of 4 HLA-gene loci (HLA-A, -B, -C, -DRB1)
4. Use of umbilical cord blood as stem-cell source
5. Use of ex vivo T-cell–depleted grafts
6. At least Grade 2 graft versus host disease (GVHD), requiring the use of systemic corticosteroids (≥1 mg/kg/day of prednisone or equivalent dose of another corticosteroid)

Protocol Amendments

There were nine global amendments to the original protocol (dated 4 August 2016). Those considered of greatest importance after trial commencement are detailed below. None of the protocol amendments performed during the course of the study were driven by data accumulated in this study, nor were there any changes that could potentially have impacted the reliability and integrity of the study results.

<u>5 February 2019</u>

Based on feedback from the Data Monitoring Committee and Regulatory Agency, eligibility criteria for viral load were expanded to allow patients meeting certain CMV infection high-risk criteria to enroll with CMV DNA ≥455 IU/mL in plasma or ≥1365 IU/mL in whole blood, to align with current clinical treatment patterns.

- A third viral-load stratum added for patients with very low viral load (CMV DNA ≥1365 IU/mL to <2730 IU/mL in whole blood or ≥455 IU/mL to <910 IU/mL in plasma at baseline) and high-risk infection, in addition to the existing low viral-load (CMV DNA ≥2730 IU/mL to <27 300 IU/mL in whole blood or ≥910 IU/mL to <9100 IU/mL in plasma) and high viral-load (CMV DNA ≥27 300 IU/mL in whole blood or ≥9100 IU/mL in plasma) strata.
- Added exclusion for concomitant letermovir and specified required washout period, to coincide with the commercial availability of letermovir.

7 December 2020

- Amends to the protocol to maintain patient safety, confidentiality, and study integrity in the context of healthcare delivery challenges presented by the coronavirus disease 2019 (COVID-19) pandemic.
- Provided flexibility to patients to opt for home healthcare solutions as permitted by local regulations. This “hybrid study design” offered patients the option of in-clinic or at-home healthcare for all study visits in the treatment phase
- Guidance was provided regarding changes to the study procedures that could be implemented for patients or study sites affected by the COVID-19 Public Health Emergency. The guidance took references from the United States Food and Drug Administration Guidance on Conduct of Clinical Trials of Medical Products during COVID-19 Public Health Emergency – Guidance for Industry, Investigators, and Institutional Review Boards, March 2020, updated 3 June 2020, and the European Medicines Agency Guidance on the Management of Clinical Trials During the COVID-19 (Coronavirus) Pandemic, Version 3 (28 April 2020).

Additional Endpoints

Additional secondary endpoints included maintenance of CMV viremia clearance with no clinical findings of tissue-invasive disease achieved at the end of week 8 through weeks 12 and 20, and recurrence of confirmed CMV viremia when patients were on or off treatment. The incidence of Grade 3 (absolute neutrophil count [ANC] $<1000/mm^3$) or Grade 4 (ANC $<500/mm^3$) neutropenia, according to National Cancer Institute Common Terminology Criteria for Adverse Events grading, while on treatment, was assessed.

Exploratory evaluations included all-cause mortality and tissue-invasive disease over the entire study period (regardless of the use of alternative anti-CMV treatment), maribavir CMV resistance profile, and treatment effect on health resource utilization. Acute care hospital stay(s) and reasons, such as treatment of acute or chronic GVHD/tissue-invasive CMV disease/neutropenia/infections, were assessed during the on-treatment and follow-up phases.

Trial Monitoring and Follow-up

During the planned period of study drug administration (8 weeks), repeat quantitative polymerase chain reaction (qPCR) testing on plasma for CMV DNA was performed by a central laboratory using COBAS® AmpliPrep/COBAS® TaqMan® CMV Test. The central laboratory plasma CMV DNA results were reported to the investigator at each trial site in

real-time. Additional qPCR testing at a local laboratory could also be performed at the investigator's discretion. Both results could be used to start treatment. CMV genotyping and virus susceptibility testing were concluded at a central laboratory for patients failing to clear viremia or experiencing recurrent viremia. During the follow-up phase of the trial (between 8 and 18 weeks after start of trial) when patients were off study drug, qPCR testing was performed on plasma by the central laboratory every 1 to 2 weeks.

Statistical Analyses

For sample size calculation, it was assumed that 68% and 60% of patients in the maribavir and valganciclovir arms, respectively, would achieve the primary endpoint. It was then estimated that to achieve >90% power to declare non-inferiority of maribavir to valganciclovir for the primary endpoint, 494 eligible patients (247 per treatment arm) were required. Considering a 10% dropout rate, 550 patients (275 patients per treatment arm) were to be enrolled and randomized.

A post hoc analysis of the proportion of patients with confirmed viremia clearance by study week for the two arms was performed by calculating the proportion of patients with CMV DNA below the lower limit of quantification at that visit and the consecutive prior visit (i.e., confirmed viremia clearance).

Supplementary Results

All-cause mortality

Frequent causes of death in the maribavir versus valganciclovir arms were GVHD (maribavir, 6 patients; valganciclovir, 0 patient), relapse of leukemia (maribavir, 2 patients; valganciclovir, 2 patients), and septic shock (maribavir, 3 patients; valganciclovir, 0 patient). Death related to CMV infection occurred in 1 patient each in the maribavir arm (CMV colitis) and valganciclovir arm (CMV pneumonia).

Supplementary Table 1. Treatment-Emergent Adverse Events Leading to Death During the On-Treatment Phase by System Organ Class, Preferred Term, and Treatment Arm (Safety Population)

**System Organ Class**<br>**Preferred Term**	**Maribavir (N = 273) n (%)**	**Valganciclovir (N = 274) n (%)**
Any TEAE leading to death	**18 (6.6)**	**12 (4.4)**
**Cardiac disorders**	**0**	**1 (0.4)**
Arrhythmia	0	1 (0.4)
**Immune system disorders**	**6 (2.2)**	**1 (0.4)**
Acute GVHD in intestine	6 (2.2)	0
Chronic GVHD in intestine	0	1 (0.4)
**Infections and infestations**	**7 (2.6)**	**5 (1.8)**
Cytomegalovirus colitis	1 (0.4)	0
Disseminated toxoplasmosis	1 (0.4)	0
Epstein-Barr virus infection	1 (0.4)	0
Fournier's gangrene	0	1 (0.4)
Fungal sepsis	0	1 (0.4)
Lower respiratory tract infection fungal	0	1 (0.4)
Pneumonia	1 (0.4)	1 (0.4)
Pneumonia cytomegaloviral	0	1 (0.4)
Septic shock	3 (1.1)	0
**Injury, poisoning, and procedural complications**	**1 (0.4)**	**0**
Subdural hemorrhage	1 (0.4)	0
**Neoplasms benign, malignant, and unspecified**	**5 (1.8)**	**3 (1.1)**
Acute myeloid leukemia	1 (0.4)	0
Acute myeloid leukemia recurrent	2 (0.7)	2 (0.7)
Diffuse large B-cell lymphoma	1 (0.4)	0
Lymphoma	1 (0.4)	0
Myelodysplastic syndrome	0	1 (0.4)

Percentages are based on the number of patients in the Safety Population within each column. Patients were counted once per System Organ Class and once per Preferred Term per treatment. The on-treatment phase started at the time of study treatment initiation through 7 days after the last dose of study treatment, or until the non-study CMV treatment initiation, whichever was earlier. TEAEs were defined as any adverse event occurring during the on-treatment phase. Adverse events were coded using MedDRA, Version 23.0.

CMV, cytomegalovirus; GVHD, graft versus host disease; MedDRA, Medical Dictionary for Regulatory Activities; N, number of patients in a treatment arm; n, number of patients experiencing the event; TEAE, treatment-emergent adverse event.

Supplementary Table 2. Identified Treatment-Emergent[a] Known or Suspected RASs to Conventional Anti-CMV Therapies[b]

	Associated resistance	Valganciclovir (N=274) n (%)	Maribavir (N=273) n (%)
Single GCV/FOS/CDV RAS in only pUL97			
**M460I**	GCV	1 (0.4)	0
**M460V**	GCV	1 (0.4)	0
C480F	GCV (also MBV)	0	1 (0.4)
C480R	GCV (also MBV)	1 (0.4)	0
**H520Q**	GCV	1 (0.4)	0
C592G	GCV	0	1 (0.4)
**A594V**	GCV	2 (0.7)	0
**L595S**	GCV	1 (0.4)	0
Multiple GCV/FOS/CDV RAS in only pUL97			
A594P+**C603W**	Ganciclovir	1 (0.4)	0

RASs in bold are those identified in the valganciclovir group and considered pUL97 canonical RASs to ganciclovir; RASs in italics are those identified in the maribavir group and considered pUL97 canonical RASs to maribavir; RASs underlined are those identified in the maribavir group and considered pUL97 canonical RASs to ganciclovir.

[a]Occurring after treatment and not present before treatment. Includes patients who did not have baseline genotyping results and had a post-transplant genotyping assessment showing resistance.

[b]CMV genotyping at baseline for RASs to conventional anti-CMV therapies was performed successfully for 508/547 subjects (92.9%). The remaining baseline samples could not be successfully genotyped for reasons that may have included polymorphisms within one of the primer binding sites, insufficient viral load, and/or the presence of polymerase chain reaction inhibitors in the sample.

CDV, cidofovir; CMV, cytomegalovirus; FOS, foscarnet; GCV, ganciclovir; MBV, maribavir; RAS, resistance-associated amino acid substitution.

Supplementary Table 3. Identified Treatment-Emergent[a] Known or Suspected RASs to Maribavir[b]

	Valganciclovir (N=274) n (%)	Maribavir (N=273) n (%)
Single maribavir RAS in only pUL97		
T409M	0	12 (4.4)
H411Y	0	6 (2.2)
Multiple maribavir RASs in only pUL97		
T409M+H411Y	0	5 (1.8)
T409M+C480F	0	1 (0.4)
T409M+C480R	1 (0.4)	0

[a]Occurring after treatment and not present before treatment. Includes patients who did not have baseline genotyping results and had a post-transplant genotyping assessment showing resistance.

[b]CMV genotyping at baseline for RASs to maribavir was performed successfully for 507/547 subjects (92.7%). The remaining baseline samples could not be successfully genotyped for reasons that may have included polymorphisms within one of the primer binding sites, insufficient viral load, and/or the presence of polymerase chain reaction inhibitors in the sample.

RAS, resistance-associated amino acid substitution; WT, wild type.

Supplementary Figure 1. Subgroup analysis of confirmed CMV viremia clearance with no clinical findings of tissue-invasive disease at week 8 with maintenance of this treatment effect through week 16, by treatment arm.

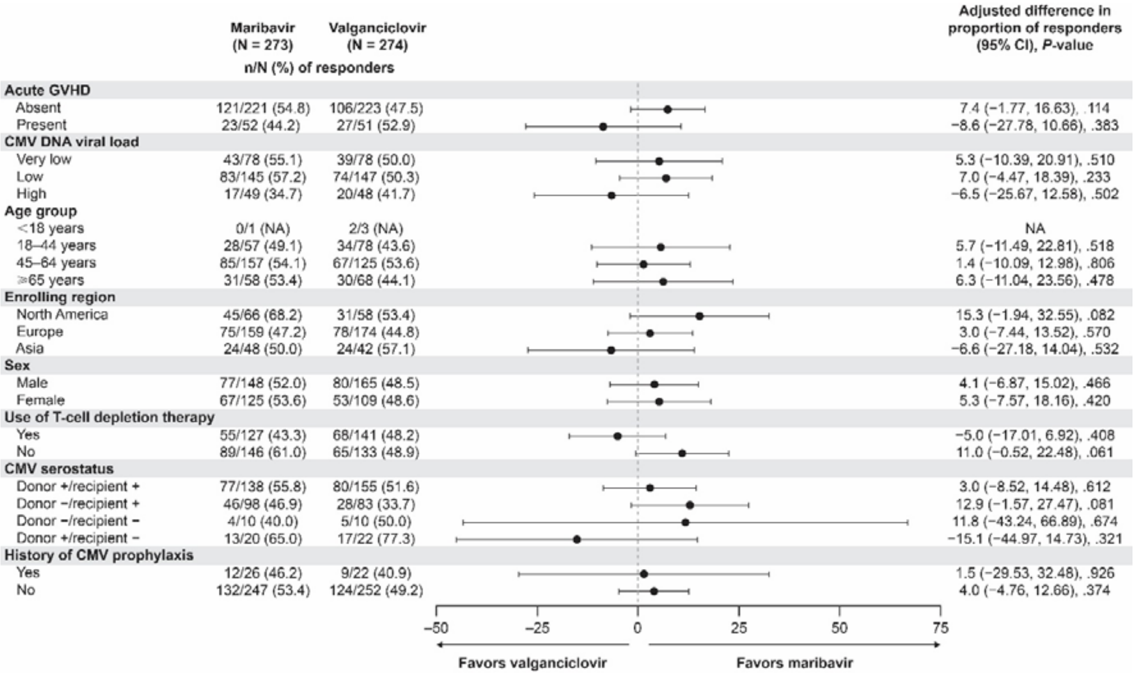

CI, confidence interval; CMV, cytomegalovirus; GVHD, graft versus host disease; N, number of patients in a treatment arm; n, number of responders; NA, not applicable.
